# Clickable Albumin Nanoparticles for Pretargeted Drug Delivery toward PD-L1 Overexpressing Tumors in Combination Immunotherapy

**DOI:** 10.1021/acs.bioconjchem.2c00087

**Published:** 2022-04-28

**Authors:** Christoph Gerke, Irene Zabala Gutierrez, Diego Méndez-González, M. Carmen Iglesias-de la Cruz, Francisca Mulero, Daniel Jaque, Jorge Rubio-Retama

**Affiliations:** † Department of Chemistry in Pharmaceutical Sciences, Faculty of Pharmacy, Complutense University of Madrid, 28040 Madrid, Spain; ‡ Ramón y Cajal Institute for Health Research (IRYCIS), Ctra. Colmenar Viejo, 28034 Madrid, Spain; § Nanomaterials for Bioimaging Group, Departamento de Fisiología, Facultad de Medicina, Universidad Autónoma de Madrid, Avda. Arzobispo Morcillo 2, 28029 Madrid, Spain; ∥ Molecular Imaging Unit, Spanish National Cancer Research Centre (CNIO), C. de Melchor Fernández Almagro 3, 28029 Madrid, Spain; @ Departamento de Física de Materiales, Facultad de Ciencias, Universidad Autónoma de Madrid, 28049 Madrid, Spain

## Abstract

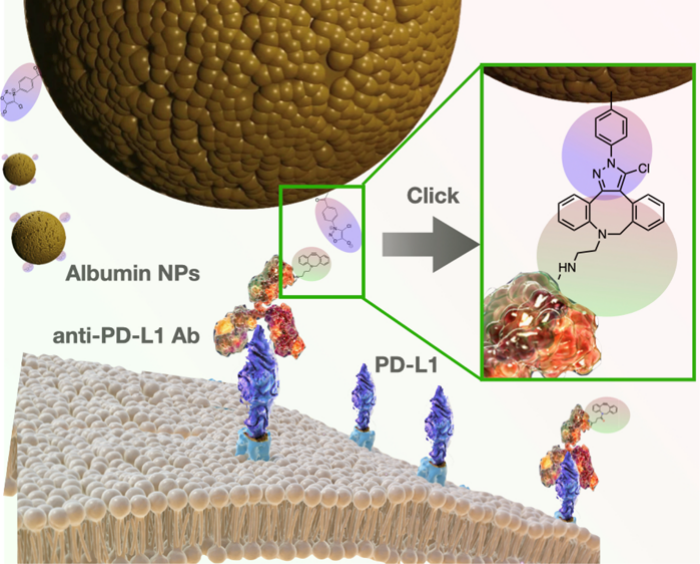

We present a simple methodology to design a pretargeted drug delivery system, based on clickable anti-programmed death ligand 1 (anti-PD-L1) antibodies (Abs) and clickable bovine serum albumin (BSA) nanoparticles (NPs). Pretargeted drug delivery is based on the decoupling of a targeting moiety and a drug-delivering vector which can then react in vivo after separate injections. This may be key to achieve active targeting of drug-delivering NPs toward cancerous tissue. In pretargeted approaches, drug-delivering NPs were observed to accumulate in a higher amount in the targeted tissue due to shielding-related enhanced blood circulation and size-related enhanced tissue penetration. In this work, BSA NPs were produced using the solvent precipitation methodology that renders colloidally stable NPs, which were subsequently functionalized with a clickable moiety based on chlorosydnone (Cl-Syd). Those reactive groups are able to specifically react with dibenzocyclooctyne (DBCO) groups in a click-type fashion, reaching second-order reaction rate constants as high as 1.9 M^–1^·s^–1^, which makes this reaction highly suitable for in vivo applications. The presence of reactive Cl-Syd was demonstrated by reacting the functionalized NPs with a DBCO-modified sulfo-cyanine-5 dye. With this reaction, it was possible to infer the number of reactive moieties per NPs. Finally, and with the aim of demonstrating the suitability of this system to be used in pretargeted strategies, functionalized fluorescent NPs were used to label H358 cells with a clickable anti-PD-L1 Ab, applying the reaction between Cl-Syd and DBCO as corresponding clickable groups. The results of these experiments demonstrate the bio-orthogonality of the system to perform the reaction in vitro, in a period as short as 15 min.

## Introduction

The development of innovative approaches for targeted therapy (drug delivery) of selective areas is of utter importance in current biomedical and clinical research, especially for oncology. The cytotoxicity of most drugs applied in chemotherapy limits the amount of the drug which can be administrated, potentially resulting in an ineffective concentration in the diseased tumor tissue. Furthermore, they cause severe adverse effects, affecting patients’ quality of life during chemotherapy.^1,2^ These two aspects make a targeted application of such drugs highly desirable. Despite the efforts in recent years and numerous publications in this area, only very few approaches succeed the translation into clinics. Especially, targeted therapies based on nanoparticles (NPs) are still falling well short of their expectations with only a very small number of FDA-approved treatments in oncology.^3^ Most established NP-based drug formulations are Doxil (liposomal doxorubicin)^4^ and Abraxane (albumin NP bound paclitaxel).^5^ Despite a significant reduction of side effects, both these types of formulations solely make use of a less effective passive (EPR-based) accumulation in the tumoral tissue. An active targeting (receptor-mediated targeting) of tumors using NP-based formulations is currently not approved by the FDA. A possibility to overcome this current lack of clinical translation could be the combination of cancer-specific antibodies (Abs), especially those already established in clinics, such as anti-human epidermal growth factor receptor 2 or anti-programmed death ligand 1 (anti-PD-L1) Abs, with NP-based drug-delivering vectors. Direct conjugation of the Abs to the NPs showed to be not an optimal option, resulting in many cases in a similar or in an even lower tumor accumulation compared to a passive accumulation of the non-functionalized NPs in comparison to their Ab-functionalized counterparts.^6,7^ A possible explanation is the drastic increase in the hydrodynamic diameter of the NP size once they are functionalized with Abs or even with Fab and F(ab)_2_ fragments, which then affects their distribution or EPR-based tumor uptake. The second explanation is the enhanced recognition of Ab-functionalized NPs by the reticuloendothelial system (RES). In fact, some reports suggest that properly shielded NPs can feature longer circulation times compared to Ab-functionalized NPs.^8,9^

**Scheme 1:**
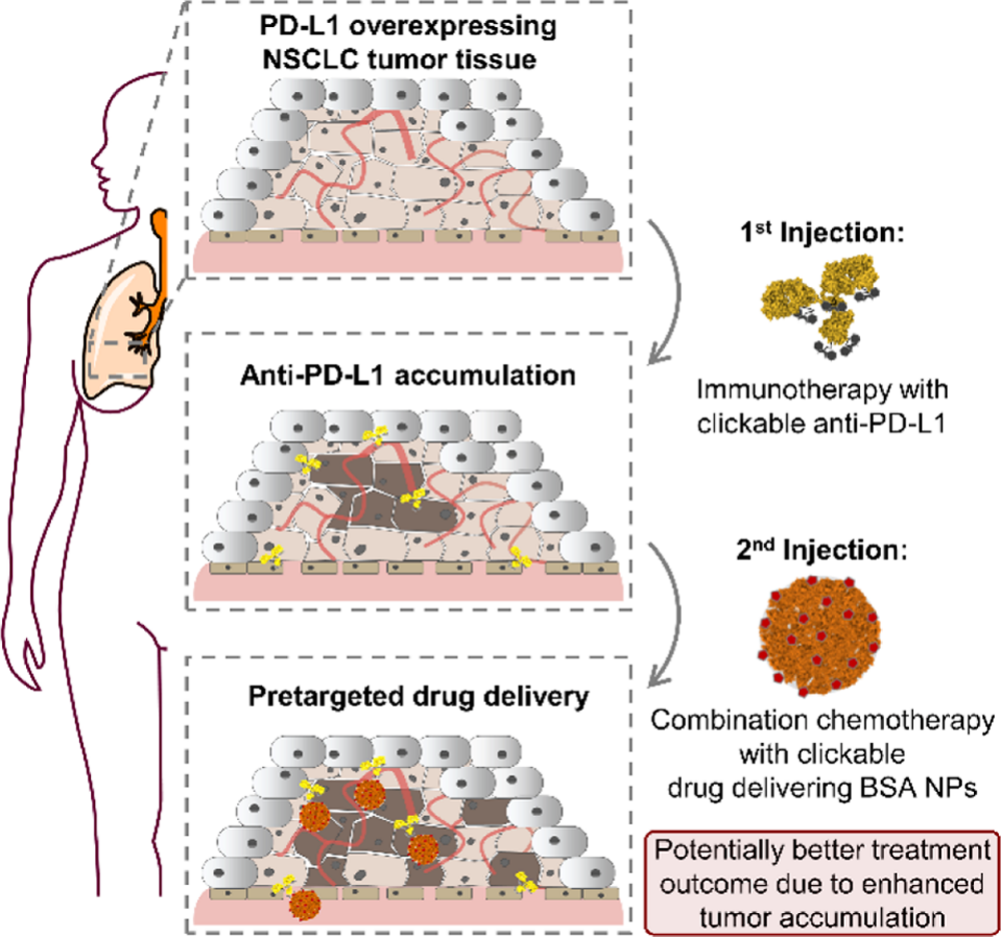
Overview of the Pretargeted Drug-Delivering Approach toward PD-L1 Overexpressing NSCLC Tumor Tissue, Applying Two Subsequent Injections of Clickable anti-PD-L1 Antibodies and Clickable BSA NPs

A possible solution that is showing promising results in primary in vitro and in vivo tests is the so-called pretargeted drug delivery. In pretargeted approaches, an active targeting molecule, such as an Ab or a peptide, is previously injected into an organism, resulting in an abundant accumulation at the targeted site. After its accumulation, a drug-delivering system, which is known to be well shielded from the RES and thus has a long circulation time, is subsequently injected.^8,9^ Both parts, the targeting molecule and the drug-delivering system, expose reactive functional groups, which can undergo a covalent conjugation within the living organism. A pretargeted drug delivery approach is schematically shown in Scheme 1. The applied reactions must be highly bio-orthogonal and fast proceeding and high yielding, even at low concentrations and in presence of complex biological media. In this vein, different types of click reactions have been developed, which are suitable for in vitro and even in vivo applications.^10,11^ Researchers in the group of Bertozzi were pioneers in this field, applying for the first time the reaction between azide and dibenzocyclooctyne (DBCO) in a living cell, thereby facilitating its later implementation inside a living organism.^12^ Reaction kinetics were, however, not optimal for in vivo application, reaching second-order reaction rate constants of 10^–3^ to 10^–2^ M^–1^ s^–1^, depending on the used DBCO derivative.^10,13,14^ Most prominent click reaction for in vivo applications is the inverse electron demanding Diels–Alder reaction between *trans*-cyclooctene (TCO) with tetrazines, reaching more suitable second-order reaction rate constants of 10^3^ to 10^4^ M^–1^ s^–1^. The first clinical phase I study of click to release TCO-based prodrugs was approved by the FDA in 2019, releasing chemotherapeutic drugs at the tumor site upon reaction with different tetrazine derivatives.^11,15^ Despite the broad application of TCO and tetrazines, a major disadvantage is the instability of TCO, which can undergo isomerization to the more stable but almost unreactive *cis*-cyclooctene (CCO) isomer, for example, under UV irradiation.^16^ Most suitable alternative, reaching reaction rates in a similar order (10^1^ to 10^2^ M^–1^ s^–1^) but providing better stability, is the strain-promoted sydnone–alkyne cycloaddition (SPSAC) between chlorosydnone (Cl-Syd) derivatives and DBCO, developed by the groups of Specklin and Taran.^17^

Here, we now present a proof-of-concept study of a potential Cl-Syd–DBCO-based pretargeted drug delivery system, combining well-studied biocompatible elements, similar to those applied in FDA-approved formulations in oncology therapy, thereby paving the way to an active drug delivery formulation based on NPs.

As active targeting moiety, anti-PD-L1 (B7-H1) Abs will be applied and functionalized with clickable DBCO moieties. Anti-PD-L1 Abs are widely applied in immunotherapy as checkpoint inhibitors, especially in non-small cell lung cancer (NSCLC) but also as treatment in other types of cancer, as proven by the three FDA-approved PD-L1 inhibiting Abs currently available in the market.^18−20^ The therapy is based on the inhibition of the PD-L1 receptor, upregulated in the microenvironment of multiple types of cancers. PD-L1 overexpression results in the deactivation of tumor-infiltrating lymphocytes (TILs) which, in turn, do not recognize the diseased cells and do not eliminate them. After PD-L1 inhibition, cytotoxic T cells are reactivated and are able to attack and eliminate cancer cells. Even though immunotherapy is highly effective in some patients, their overall response rate (ORR) is generally low. The ORR of PD-L1 monotherapy, for example, merely lies between 14 and 24%.^21^ Due to these unsatisfying numbers, currently, PD-L1 therapy is often combined with conventional platin-based or paclitaxel chemotherapy.^22^ We believe that the simple modification of the anti-PD-L1 Abs and albumin-based NPs, which are also used in combination therapy (anti-PD-L1 combined with Abraxane used in advanced NSCLC^21^), will be a straightforward procedure to potentially increase treatment outcomes due to a higher accumulation of drug-loaded NPs at the tumor site (see Scheme 1).

## Results and Discussion

### Synthesis and Reaction Rate Constant Determination of the Clickable Cl-Syd Derivative

**Scheme 2:**
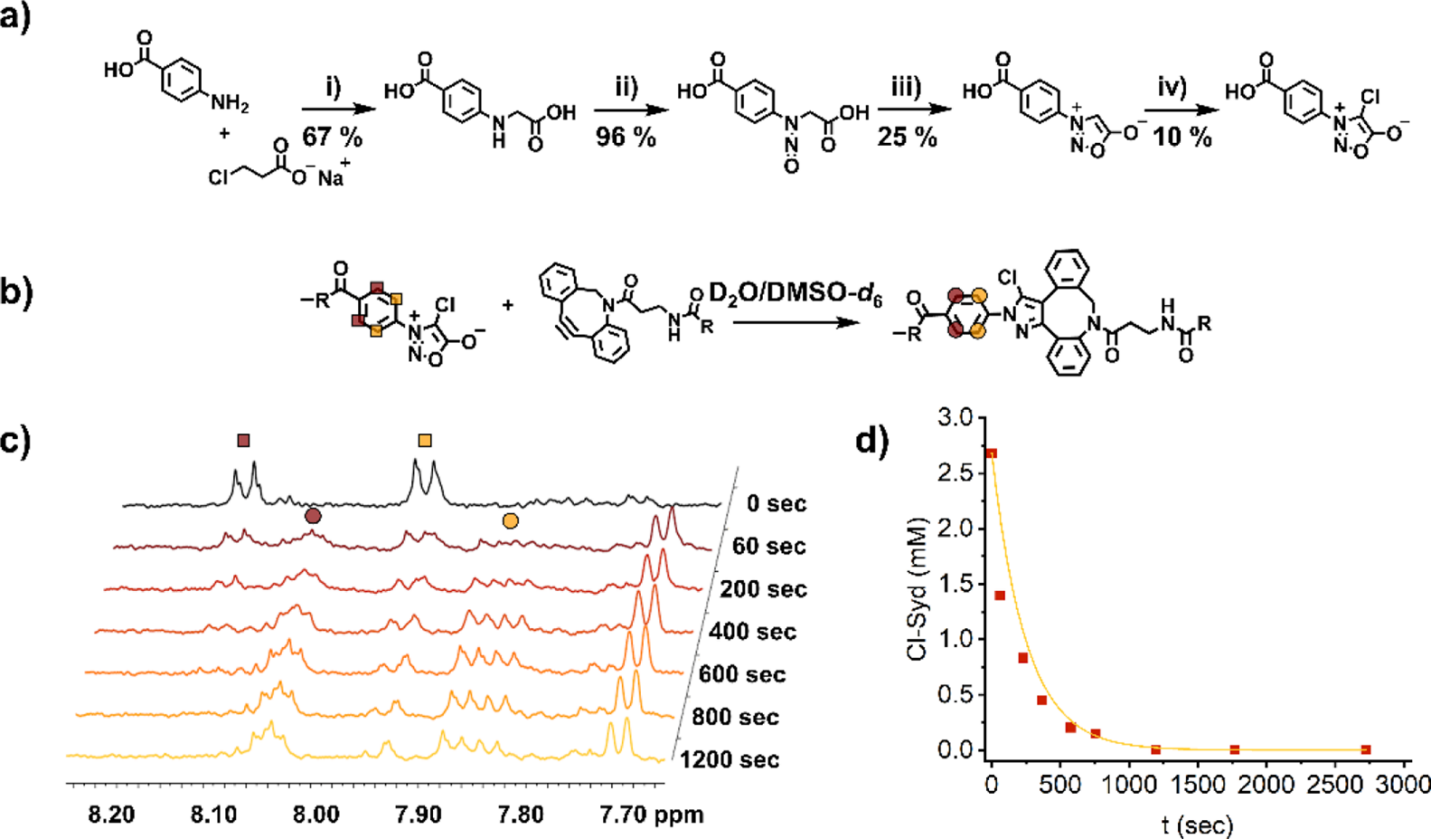
(a) Reaction Overview of *p*-Carboxyphenyl-Chlorosydnone According to the Published procedure^23^; (b) Reaction Overview of the SPSAC Click Reaction between a Cl-Syd Derivative and a DBCO Derivative; (c) ^1^H NMR (500 MHz, D_2_O/DMSO-*d*_6_) Data of the Chemical Shift Region from 8.25 until 7.65 ppm, Showing the Variation in Chemical Shift of the Signals of the Cl-Syd Neighbored Phenyl Moiety during the Course of the Reaction, without the Presence of the DBCO Derivative (0 s) as well as for Six Further Time Points between 60 and 1200 s of Reaction Time; and (d) Consumption of the Cl-Syd Derivative Over the Reaction Time with the DBCO Derivative in Excess Conditions for each reaction step of the synthesis of *p*-carboxyphenyl-chlorosydnone (i) aminobenzoic acid and sodium chloroacetate dissolved in H_2_O were refluxed overnight; (ii) addition of sodium nitrite to *p*-carboxyphenyl *N*-substituted α-amino acid in HCl (10%) at 0 °C and subsequent stirring overnight under N_2_; (iii) Baker–Ollis syndone synthesis^24^ of the *N*-nitroso derivative of *p*-carboxyphenyl N-substituted α-amino acid. The *N*-nitroso derivative was stirred in acetic acid anhydride at 100 °C for 3 h; and (iv) to a solution of *p*-carboxyphenyl-sydnone in dioxane/HCl (1 M) 2/1, sodium hypochlorite (10%) was added dropwise, and the reaction stirred for 4 h. Yields are given for each reaction step.

Other than the DBCO derivatives, which are widely used, the Cl-Syd compounds required for the stated approach are not commercially available and were thus synthesized. An adapted protocol from Kolodych and Taran was used for the synthesis of the *p*-carboxyphenyl-chlorosydnone derivative.^23^ A schematic overview of the synthesis and the structure of the final Cl-Syd are shown in Scheme 2a. Based on this protocol, the final Cl-Syd derivative was synthesized with an overall yield of 1.6% (four-step synthesis with low yielding last step, see Scheme 2a) and a purity above 88% (determined by HPLC, impurities not reactive in next amine coupling steps). Details of the synthesis and information about the characterization of the intermediates and the final compound are stated in the Supporting Information.

To verify the published second-order reaction rate constants of the here used Cl-Syd, we performed a kinetic study using a Cl-Syd and a DBCO derivative as model compounds. We deliberately did not make use of the *p*-carboxyphenyl-chlorosydnone during this kinetic study but rather used a Cl-Syd model compound with a higher molecular weight that contains an amide linkage in contrast to the free carboxylic acid group on the phenyl moiety. Thereby, factors such as size-related diffusion or steric hindrance and the influence of the free acid on the reaction rate are taken into consideration in the kinetic study, giving a more relevant value for the later application of this click reaction. After the attempt to monitor the reaction via the formation of a fluorescent product (λ_ex_: 315 and λ_em_: 385 nm), as published by the group of Taran and Specklin,^17^ a monitorization using nuclear magnetic resonance (NMR) spectroscopy was chosen since the DBCO starting material already shows a strong fluorescence at the same wavelength as the formed SPSAC click product that hampers the possibility to follow the reaction kinetics, as mentioned by Taran and Specklin.^17^ Therefore, the rate constant values given by those authors which are in the order of 10^2^ M^–1^ s^–1^ should be treated with caution.

Although no proton is directly involved in the click reaction, the course of the click reaction was able to be monitored via ^1^H NMR due to the variation in the chemical shift of the signals from the Cl-Syd neighbored phenyl moiety during the course of the reaction (8.15 and 7.95 ppm for Cl-Syd to 8.05 and 7.85 ppm in the click product in D_2_O/DMSO-*d*_6_, see Scheme 2c). The consumption of Cl-Syd, as shown in Scheme 2d, was determined using the integrals of the disappearing signals for the phenyl moiety, which was subsequently applied in the calculation of the second-order reaction rate constant. The calculated rate constant for the here used system of 1.9 M^–1^ s^–1^ was similar to the previously calculated constant for *p*-carboxyphenyl-chlorosydnone from HPLC data (1.6 M^–1^ s^–1^).^23^ Having obtained the same rate constants as published for *p*-carboxyphenyl-chlorosydnone applying HPLC analysis, it can be stated that the application of an amide-coupled Cl-Syd with higher molecular weight does not negatively affect the reaction kinetics. Although the reaction kinetics do not reach rate constants of TCO-tetrazine-based bio-orthogonal reactions, the superior chemical stability of the Cl-Syd groups in comparison with the TCO-ones, it is an interesting feature that simplifies the storage of the reactive molecules and increases the result reliability. The determined reaction rate constant is with a value of 1.9 M^–1^ s^–1^ 2 orders of magnitude larger compared to those of common SPAAC reactions using azides, which have been previously applied in vivo.^13,14^ Therefore, the successful application of this reaction for in vivo applications can be assumed with high confidence.

### Synthesis and Characterization of Clickable BSA NPs

**Figure 1:**
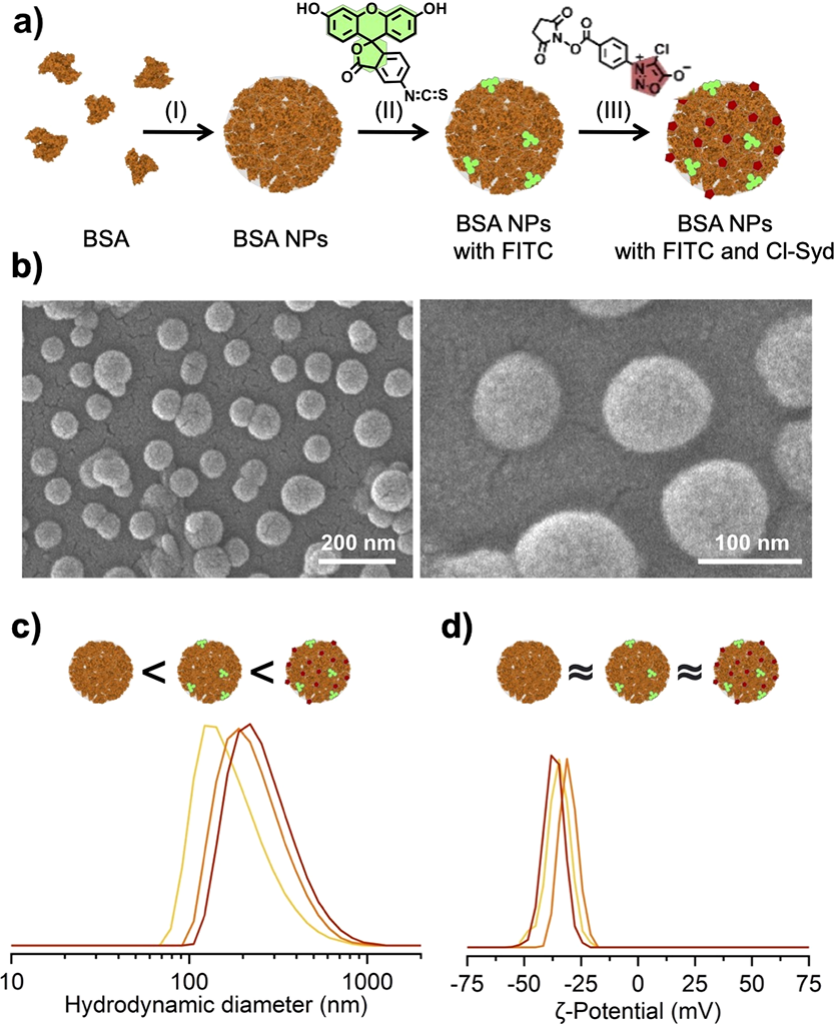
(a) Preparation overview of FITC-functionalized CS-BSA NPs with (I) being the generation of the BSA NPs by the desolvation method, (II) the functionalization of the BSA NPs with FITC, and (III) the final functionalization with the NHS ester of the here used Cl-Syd derivative; (b) SEM image of the non-functionalized BSA NPs; (c) DLS results of the non-functionalized BSA NPs (yellow) and BSA NPs functionalized with FITC (orange) and BSA NPs functionalized with FITC and Cl-Syd (red); and (d) ζ-potential of the non-functionalized BSA NPs (yellow) and BSA NPs functionalized with FITC (orange) and BSA NPs functionalized with FITC and Cl-Syd (red).

The bovine serum albumin (BSA) nanoparticles, on which the clickable Cl-Syd groups were aimed to be immobilized eventually, were generated by the desolvation method, using an established protocol from the working group of Rubio-Retama (for details, see the experimental section).^25^ The formed BSA NPs (Figure 1) were stirred overnight. After multiple purification steps by centrifugation, NPs with a hydrodynamic diameter of 140 nm were isolated. ζ-potential analysis revealed a highly negative surface charge of −35 mV, which is in accordance with stated ζ-potentials for this type of NPs prepared at pH 8.5.^25^ The BSA NPs were held in carbonate buffer with a pH 8.5 at 4 °C until further usage. After 1 month of storage at 4 °C, the NPs neither showed a change in their hydrodynamic diameter nor ζ-potential, thereby demonstrating their high stability. Besides confirming the size of the NPs, the high monodispersity of the NPs was proven by scanning electron microscopy (SEM) analysis. Results from DLS and SEM analyses of the synthesized BSA NPs are shown in Figure 1b,c.

The stable BSA NPs were subsequently functionalized with the previously synthesized Cl-Syd groups. The choice to synthesize the carboxy-benzene derivative was based on two reasons: first due to the high reactivity derived from the sydnone when bound to a phenyl group^23^ and second because the presence of the carboxylic acid group can be used directly to covalently bind the Cl-Syd to the BSA NPs by well-established amide coupling protocols. Therefore, the carboxylic acid of the Cl-Syd derivative was activated using DCC/NHS (*N*,*N*′-dicyclohexylcarbodiimide/*N*-hydroxysuccinimide) and subsequently purified, as described by the groups of Specklin and Taran.^17^ Subsequently, BSA NPs were functionalized with the NHS-activated Cl-Syd derivative, as described in the experimental section. DLS analysis revealed a slight increase in hydrodynamic diameter after functionalization but no significant change in ζ-potential (165 nm, ζ-pot. −31 mV) and good stability of the Cl-Syd-functionalized BSA NPs (CS-BSA NPs from here on) in carbonate buffer (pH 8.5). It was observed that the initial and the CS-BSA NPs were less stable in PBS buffer, even at an elevated pH of 8.0; thus, carbonate buffer (pH 8.5) was used for functionalization reactions, washing steps, and final storage until usage. The here added amounts of DMSO did not affect the stability of the BSA NPs, as investigated in control experiments (data not shown). Fourier transform infrared (FTIR) spectroscopy and ^1^H NMR analyses were ambiguous to directly prove the linkage of the Cl-Syd moieties on the NP surface.

**Figure 2:**
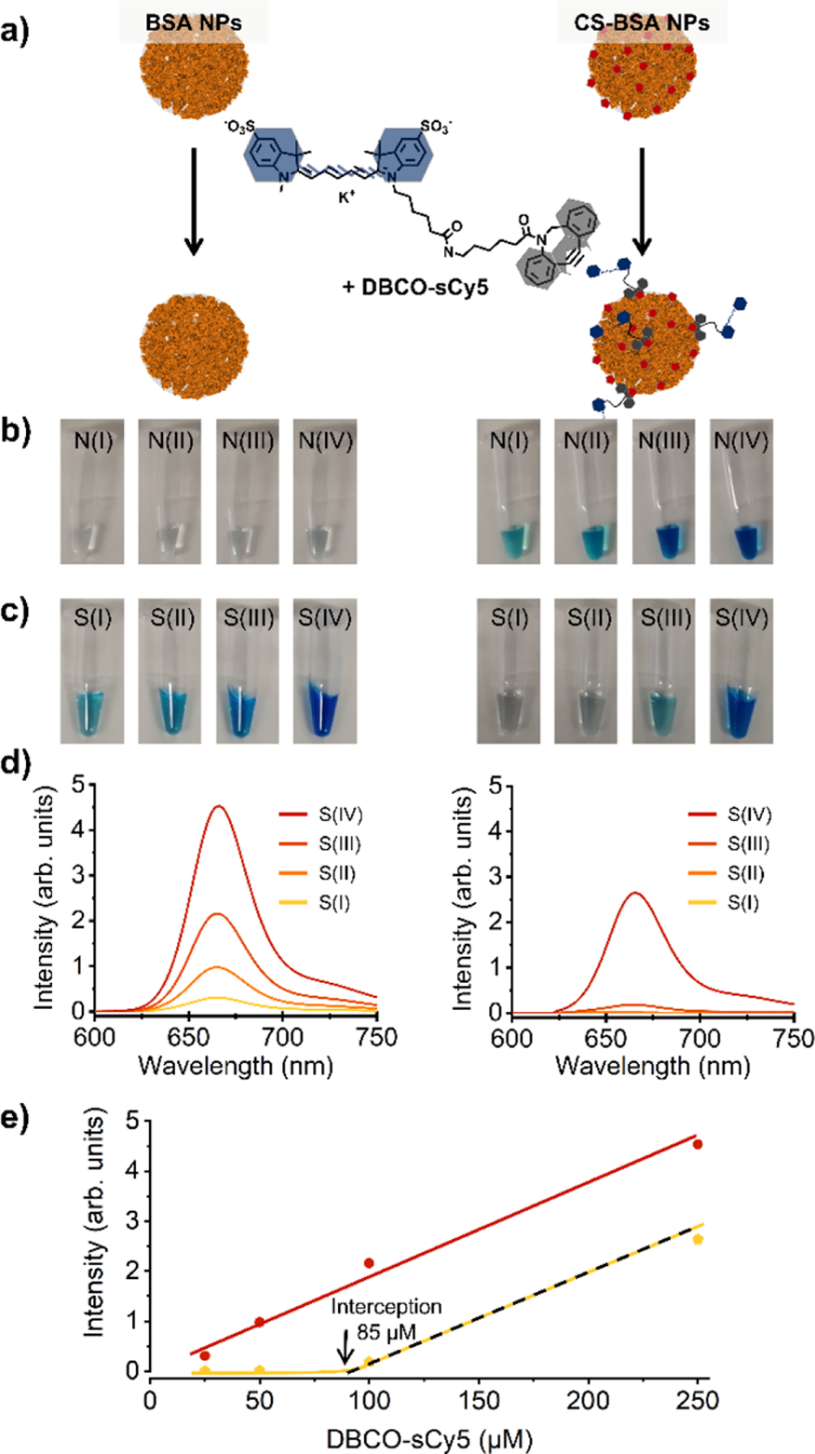
(a) Schematic overview of the experiments, including a control experiment using non-functionalized BSA NPs, which was performed to evaluate the unspecific physisorption of the DBCO-sCy5 on the surface of the BSA NPs (left), and the specific reaction between functionalized CS-BSA NPs and DBCO-sCy5, to evaluate the reactivity of the moieties; (b) images to show the aspect of the purified BSA NPs (N for nanoparticles) after incubation with DBCO-sCy5 at four different concentrations [(I): 25 μM, (II): 50 μM, (III): 100 μM, and (IV): 250 μM], showing the aspect of non-functionalized BSA NPs (control experiment) on the left and the aspect of CS-BSA NPs on the right; (c) images to show the aspect of the first supernatant removed (S for supernatant) after incubation with DBCO-sCy5 at four different concentrations [(I): 25 μM, (II): 50 μM, (III): 100 μM and (IV): 250 μM], showing the aspect of supernatant removed from non-functionalized BSA NPs (control experiment) on the left and the aspect of supernatant removed from CS-BSA NPs on the right; (d) results from fluorescent measurement of the first supernatants removed for the non-functionalized (control experiment) (left) and CS-BSA NPs (right); and (e) PL emission representation of the first supernatants obtained after incubating the DBCO-sCy5 with different concentrations of non-functionalized (control experiment) and Cl-Syd functionalize BSA, indicating the *x*-axis interception of the linear regression for the plot of sCy5 emission of the first supernatants removed from CS-BSA NPs after incubation used to calculate the amount of Cl-Syd moieties per BSA NP.

To prove the presence of fully functional clickable groups, a click reaction with a DBCO bearing cyanin dye was performed. As clickable dye, DBCO-modified sulfo-cyanine-5 dye (DBCO-sCy5) was chosen. To 100 μL of a solution of 1 mg/mL CS-BSA NPs in carbonate buffer (pH 8.5), four different amounts of DBCO-sCy5 were added (final concentrations of DBCO-sCy5 of 25, 50, 100, and 250 μM) and incubated for 4 h at RT. After excessive washing steps, the CS-BSA NPs incubated with DBCO-sCy5 showed an intense blue color, thereby proving the presence of the Cl-Syd moieties on the BSA NP surface (Figure 2). For the sake of comparison, images of non-functionalized BSA NPs, which were incubated with the same concentrations of DBCO-sCy5, are shown as well. The non-functionalized BSA NPs, also after incubation using high dye concentrations of 250 μM, did not show any coloration, thereby excluding the possibility of non-specific physisorption of the dye on the BSA NPs. By fluorescence measurements, complete consumption of the 25 μM and 50 μM sCy5-DBCO after incubation with clickable BSA NPs was observed. No remaining dye was detected in the first supernatant after CS-BSA NP centrifugation, as shown in Figure 2c,d (right). Supernatants of the non-functionalized BSA NPs incubated with a final dye concentration of 25 and 50 μM, on the other hand, show an intense blue color since no reaction between the dye and the BSA NPs occurred (Figure 2c,d, left). The photoluminescent (PL) emission of the supernatants isolated from the non-functionalized BSA NPs increases proportionally with the concentration of DBCO-sCy5 added to the solution (see red linear regression in Figure 2e). When plotting the PL emission of the supernatants removed from the incubated CS-BSA NPs, an increasing linear regression can be observed above a DBCO-sCy5 concentration of 100 μM, which runs parallel to the regression of the supernatants removed from non-functionalized BSA NPs (see orange regression in Figure 2e). The *x*-axis interception of this second linear regression can give information about the amount of clickable Cl-Syd moieties present per BSA NP since the amount of consumed DBCO-sCy5 can be directly correlated to the total amount of clickable groups on the NP surface. The DBCO-sCy5 concentration, which is required to completely saturate the CS-BSA NPs, was determined to be 85 μM, as given by the *x*-axis interception. The two linear regressions of the PL emission of separated supernatants plotted against the initial DBCO-sCy5 concentration are shown in Figure 2e, including the concentration at the *x*-axis interception. Considering that 0.1 mg/mL of BSA NPs with a hydrodynamic diameter of 120 nm corresponds to a total number of 6 × 10^12^ BSA NPs per mL (approx. 1 pmol), it can be concluded that each CS-BSA NP is functionalized with approximately 15.000 Cl-Syd moieties. A detailed description of this calculation can be found within the Supporting Information section.

### In Vitro Assays of Clickable BSA NPs and Clickable Anti-PD-L1 Abs

**Figure 3:**
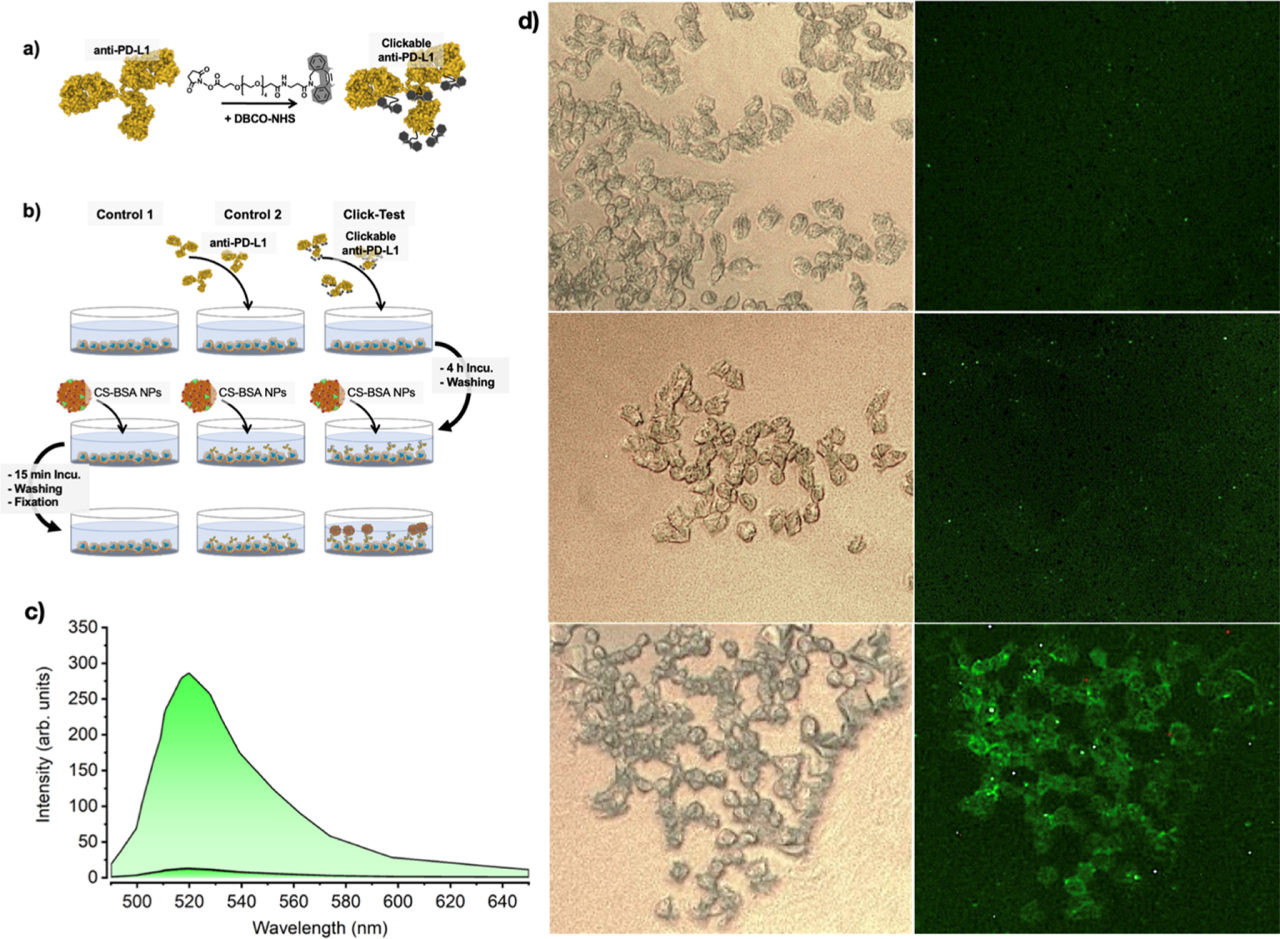
(a) Functionalization of anti-PD-L1 Ab with DBCO-NHS; (b) overview of the in vitro assay, showing the first control experiment without addition of an Ab (left), the second control experiment with the addition of non-functionalized anti-PD-L1 (center) and the experiment using the clickable anti-PD-L1 Ab, resulting in the accumulation of CS-BS NPs on the surface of H358 cells (right); (c) PL emission spectra obtained from the cell cultures used in the control 1, control 2, and the pretargeted reaction named as the click test; and (d) microscopy results including the optical images of the H358 cells (left) and the fluorescent images of the same cells (right) showing a strong fluorescent signal on the H358 cells previously treated with clickable anti-PD-L1 Abs (bottom right).

After proving the successful coupling of the clickable groups on the NP surface, an in vitro assay was performed to demonstrate the potential of the DBCO-anti-PD-L1/CS-BSA NP system for pretargeted drug delivery in lung cancer. As cell line, the human NSCLC cell line H358 was chosen since anti-PD-L1 therapy, and its combination with Abraxane is an FDA-approved therapy and widely used for treatment of this type of lung cancer.^21^ Furthermore, PD-L1 overexpression in H358 cells line has been confirmed previously.^26^ For the assay, clickable anti-PD-L1 Abs and clickable BSA NPs, which can be visualized by a fluorescent microscope or a well-plate reader, were required. Since the Cl-Syd moieties were coupled to the BSA NPs, the corresponding reactive DBCO groups for the SPSAC click reaction were covalently bound to the anti-PD-L1 Abs. The anti-PD-L1 Abs were functionalized with a DBCO derivative bearing an activated NHS-ester, allowing for its direct coupling to primary amine groups of the Abs. Furthermore, the DBCO derivative contained a short polyethylene glycol spacer of four repeating units (PEG_4_), thereby ensuring flexibility and spacing between the clickable groups and the targeting Ab (see Figure 3a). The detailed Ab functionalization conditions are given in the experimental section. Using an anti-PD-L1 to DBCO-PEG_4_-NHS ratio of 1:5 for the functionalization of primary amine groups of the Ab, an introduction between 2 and 3 DBCO moieties can be expected as observed in earlier studies applying similar coupling procedures.^27^

For CS-BSA NP visualization, the NPs were functionalized with a suitable dye. Fluorescein was used as fluorescent dye, which was coupled to primary amine groups of the BSA as isothiocyanate derivative [fluorescein isothiocyanate (FITC)]. The initial BSA NPs were first functionalized by FITC, followed by the introduction of the Cl-Syd click groups, applying the protocol stated earlier (see the Experimental Section for both coupling procedures). Each functionalization step was monitored by DLS and ζ-potential measurements (results shown in Figure 1c,d). Again, a slight increase in the hydrodynamic diameter of the BSA NPs was observed after each coupling procedure. Hydrodynamic diameter was found to be 190 and 220 nm for FITC and FITC + Cl-Syd-functionalized BSA NPs, respectively (see Figure 1c,d). The ζ-potential was not significantly affected by the functionalization of the BSA NPs and resulted in a value of −32 mV after FITC coupling and −36 mV after the subsequent Cl-Syd introduction. Besides the morphological analysis, also the fluorescence of the BSA NPs after FITC and Cl-Syd coupling was measured. For both types of BSA NPs, FITC, and FITC + Cl-Syd bearing NPs, a strong emission at 525 nm after excitation at 480 nm was observed, proving the successful incorporation of FITC groups on the surface of the BSA NPs.

The developed in vitro assay included three different sets of experiments, two of which being control experiments either lacking the addition of Ab or using non-functionalized anti-PD-L1 Ab. The in vitro assay was performed as follows: first, H358 cells were transferred into wells of a 24-well-plate and cultured for 24 h. In the second step, some wells were incubated with the DBCO-functionalized anti-PD-L1 Abs (click-test, see Figure 3b), whereas other wells were incubated with non-functionalized anti-PD-L1 Abs (control 2, see Figure 3b). In both cases, 2.5 μg of Abs was added into each well and incubated for 4 h at 37 °C, allowing the anti-PD-L1 Abs to interact and bind to the overexpressed PD-L1 on the cell surface of the H358 cells. Into an additional two wells, neither functionalized nor non-functionalized anti-PD-L1 were added (control 1, see Figure 3b). After the Ab incubation steps, all wells were washed three times with PBS buffer, and new PBS buffer (pH 7.4) was added. As the next step, 50 μg of FITC-functionalized CS-BSA NPs were added to all wells and incubated for 15 min to see the immediate reaction of the clickable NPs with H358 cell-adhered clickable anti-PD-L1. Thereby, only a very short interaction between the two clickable counterparts is allowed, mimicking a brief encounter in the organism. Each experiment (control 1, control 2, and click test, see Figure 3) was performed in duplicate. After incubation, the wells were extensively washed, and cells were subsequently fixed using a 2% paraformaldehyde solution in PBS.

Once fixed, the cells were analyzed using a fluorescent microscope coupled to a spectrometer. In the two control experiments, no fluorescent signal of the FITC and thus no accumulation of the CS-BSA NPs can be observed after an incubation time of 15 min (see Figure 3c). In the set in which the clickable anti-PD-L1 Ab was used, a significant accumulation of CS-BSA NPs around the H358 cells was observed, giving a signal 24-fold higher than the obtained signal from the control experiments (see Figure 3d). This result proves the successful coupling of the CS-BSA NPs to the clickable Abs and the bio-orthogonality of the reaction on the pretargeted cells after short incubation times. Since no accumulation of CS-BSA NPs was observed in the control experiments, a highly specific accumulation, only once DBCO moieties are present on the H358 cell surface, was proven. In the experiments that lacked DBCO, no unspecific binding can be seen after 15 min incubation time.

## Conclusions

In this work, BSA NPs with a mean hydrodynamic diameter of 120 nm and ζ-potential of −36 mV were synthesized, which have been functionalized with clickable moieties based on Cl-Syd. This molecule confers pretargeting properties to the BSA NPs that could be used to accumulate them in a specific area that has been previously targeted by an Ab equipped with a corresponding clickable DBCO group. The previously reported second-order reaction rate constant of 2 M^–1^·s^–1^ was confirmed for the here used Cl-Syd DBCO click pair, monitoring the reaction by NMR spectroscopy. The concept of pretargeted drug delivery has been tested in two ways: first, a click reaction between CS-BSA NPs and a DBCO-functionalized sCy5 was performed to determine the performance of the system in conditions with low steric hindrance; this experiment permitted to infer the number of reactive moieties existing on the surface of the BSA NPs which determined to be approximately 15.000 molecules per NP. Second, the click reaction was carried out in an in vitro experiment, between clickable CS-BSA NPs and anti-PD-L1 Ab functionalized with DBCO, that had been previously incubated with PD-L1 overexpressing H358 cells. The results of these experiments demonstrate the suitability of this click reaction to perform the pretargeting strategy in vitro between two subunits, BSA NPs and anti-PD-L1 Abs, which are not exempt from steric hindrance using incubation times as short as 15 min. From the encouraging results of this work on pretargeting of CS-BSA NPs combined with the use of anti-PD-L1 Ab as the pretargeting moiety, a well-established FDA-approved checkpoint inhibitor, we realize the bright future of this and similar platforms to achieve simultaneous immuno- and pretargeted chemotherapy of different cancers that overexpress PD-L1. In ongoing studies, the here described system will be tested in vivo, thoroughly evaluating the biodistribution of the two clickable components and their successful reaction inside a living organism. Furthermore, paclitaxel encapsulated CS-BSA NPs will be prepared, and a potential improvement of a pretargeted combinational immunotherapy in comparison to a common combinational immunotherapy using anti-PD-L1 and Abraxane will be examined.

## Experimental Section

### Synthesis of BSA NPs

The BSA NPs were synthesized by the desolvation methods, applying an established protocol from the group of Rubio-Retama.^25^ In short, to a solution of 5 mg of BSA in 1 mL of carbonate buffer (20 mM, pH of 8.5), 4 mL of ethanol was added at a flow rate of 3 mL/min. Upon addition of ethanol, the BSA NPs precipitated, and 11.5 μL of glutaraldehyde (0.25 wt %) was added for BSA cross-linkage, thereby ensuring their stability.

### Functionalization of BSA NPs with NHS-Activated Cl-Syd

To functionalize the BSA NPs with clickable Cl-Syd groups, 50 μL of an NHS-activated Cl-Syd stock in DMSO (30 mM) was added to a suspension of BSA NPs at a concentration of 1 mg/mL in carbonate buffer (pH 8.5). The reaction was stirred overnight and subsequently purified by centrifugation. The BSA NPs were washed three times with carbonate buffer.

### Functionalization of BSA NPs with FITC

For FITC introduction, 50 μL of a FITC stock in DMSO (6.4 mM) was added to 1 mg/mL BSA NPs in carbonate buffer (pH 8.5). The reaction was allowed to proceed for 3 h at RT. FITC-functionalized BSA NPs were purified by three washing steps using carbonate buffer.

### Anti-PD-L1 Functionalization with Clickable DBCO Moieties

For the Ab functionalization, 5 μL of DBCO-PEG_4_-NHS stock (2.2 mM) in DMSO was added to a solution of 330 μg of anti-PD-L1 in 200 μL of PBS (pH 8.0) and incubated for 4 h at RT. After the coupling, the DBCO-functionalized Abs were purified by centrifugal concentrators with a molecular weight cutoff of 50 kDa.

## Supporting Information Available

Materials and instrumentations used, analytical data about the synthesized Cl-Syd moiety, and data about the performed kinetic NMR and information about the calculation to determine the degree of functionalization of BSA NPs (PDF)

## Abbreviations

Ab: antibody
BSA: bovine serum albumin
CCO: *cis*-cyclooctene
Cl-Syd: chlorosydnone
DBCO: dibenzocyclooctyne
DCC: *N*,*N*′-dicyclohexylcarbodiimide
EPR: enhanced permeability and retention
FDA: food and drug administration
FITC: fluorescein isothiocyanate
HER: human epidermal growth factor receptor 2
HPLC: high-pressure liquid chromatography
IEDDA: inverse electron demanding Diels–Alder
IR: infrared
MWCO: molecular weight cutoff
NHS: *N*-hydroxysuccinimide
NMR: nuclear magnetic resonance
NP: nanoparticle
NSCLC: non-small cell lung cancer
ORR: overall response rate
PBS: phosphate-buffered saline
PD-L1: programmed cell death 1 ligand 1
PEG: polyethylene glycol
sCy5: sulfo-cyanine-5 dye
Syd: sydnone
TCO: *trans*-cyclooctene
TIL: tumor-infiltrating lymphocytes
RES: reticuloendothelial system
SPSAC: strain-promoted sydnone–alkyne cycloaddition
