# Supplementary material for: Clickable Albumin Nanoparticles for Pretargeted Drug Delivery toward PD-L1 Overexpressing Tumors in Combination Immunotherapy
Source: Bioconjug Chem. 2022 Apr 28;33(5):821–8. doi: 10.1021/acs.bioconjchem.2c00087 (PMC9121340; doi:10.1021/acs.bioconjchem.2c00087)
Supplement: Supplementary file 1 — bc2c00087_si_001.pdf [file bc2c00087_si_001.pdf]

Supporting information

Clickable albumin nanoparticles for pretargeted drug delivery towards PD-L1 overexpressing tumors in combination immunotherapy

Christoph Gerke[1,2], Irene Zabala Gutierrez[1], Diego Méndez-González[2,3], M. Carmen Iglesias-de la Cruz[2,3], Francisca Mulero[4], Daniel Jaque[2,3], Jorge Rubio-Retama[1,2]*

1 Department of Chemistry in Pharmaceutical Sciences, Faculty of Pharmacy, Complutense University of Madrid, 28040 Madrid, Spain.

2 Ramón y Cajal Institute for Health Research (IRYCIS), Ctra. Colmenar Viejo, 28034 Madrid, Spain.

3 Nanomaterials for Bioimaging Group, Department of Physiology, Faculty of Medicine, Avda. Arzobispo Morcillo 2, Autonomous University of Madrid, 28029 Madrid, Spain.

4 Molecular Imaging Unit, Spanish National Cancer Research Centre (CNIO), C. de Melchor Fernández Almagro 3, Madrid, Spain.

This supporting information includes the following sections:

1. Material
2. Instrumentation
3. Cl-Sydnone synthesis and analytical data
4. Kinetic NMR study
5. Synthesis of BSA NPs
6. Functionalization of BSA NPs with NHS activated Cl-Syd and FITC
7. Determination of Cl-Syd functionalization on BSA NPs
8. anti-PD-L1 functionalization with clickable DBCO moieties
9. References

1 Materials:

Bovin Serum Albumin (BSA), heat shock fraction, pH 5.2 (≥ 96%), glutaraldehyde (50% solution in water), 4-aminobenzoic acid (for synthesis), fluorescein isothiocyanate (FITC), sodium chloroacetate (for synthesis), sodium nitrite (for synthesis), acetic anhydride (for synthesis), sodium thiosulfate (98%), sodium hypochlorite (6-14% active chlorine, Supelco), silica gel, dicyclohexylcarbodiimide (DCC, 99%), sodium bicarbonate (for molecular biology) and PBS were purchased from Sigma Aldrich/Merck. Ethanol (96%), acetic acid (pharma grade) and sodium sulfate (anhydrous) were purchased from PanReac. Methanol (99.8%), ethyl acetate (EtOAc, 99.5%), dichloromethane (DCM, 99.8%)) and dioxane (for analysis, with 25 ppm BHT) were purchased from LabKem. Sulfo-Cyanine5 DBCO was purchased from Lumiprobe. DBCO-PEG(5)-NHS was purchased from Iris Biotech. All compounds were used without further treatment. anti-human PD-L1 (B7-H1, 6.61 mg/mL in PBS at pH 7.0) was purchased from BioXCell.

2 Instrumentation

Nuclear Magnetic Resonance spectroscopy (NMR)

^{1}H-NMR (250 MHz) were recorded on a Bruker AV 250 MHz. Chemical shifts of all NMR spectra were reported in delta (δ) expressed in parts per million (ppm). The signal of residual solvent was used as internal standard (For 1H-NMR: δ 2.50 ppm for DMSO-$_{d6}$). The following abbreviations are used to indicate the multiplicities: s, singlet; d, doublet; m multiplet. All measurements were performed at 25°C if not stated otherwise.

Reversed Phase - High Pressure Liquid Chromatography (RP-HPLC)

Measurements were performed on an Agilent 1220 Infinity II instrument coupled to a variable wavelength detector (VWD) (set to 254 nm). As HPLC column a Poroshell 120 EC-C18 (4.6×50 mm, 2.7 µm) RP Column from Agilent was used. The mobile phases A and B were H2O/ACN (95/5) and H2O/ACN (5/95), respectively. Both mobile phases contained 0.1% of formic acid. The flow rate was set to 0.5 mL/min. All compounds were analyzed using a liner gradient starting with 100% mobile phase A reaching 50% mobile phase B within 20 min. The temperature of the column compartment was set to 25 °C. Chromatogram analysis was done within the EZ Chrome software from Agilent Technologies.

Direct ESI-Mass Spectrometry

ESI-MS measurements were performed with a Bruker HCT ultra PTM Discovery system with a direct inlet via syringe pump, an ESI source and a quadrupole followed by an ion trap mass analyzer.

Dynamic Light Scattering (DLS) and ζ-potential

DLS measurements were performed on a Nano Series Zetasizer and ζ-potential measurements on a Nano-ZS Instrument both from Malvern. For DLS measurements, the accumulation time was automatically determined during each measurement. The multimodal analysis method was selected, z-average was calculated from the correlation function and the hydrodynamic diameter was derived using the Einstein-Stokes equation. ζ-potential measurements were acquired at 25 °C using the automatic mode (minimum 10 runs – maximum100 runs) with an equilibration time of 120 s and the Smoluchowski fit model. Each sample was measured three independent times and the average value was calculated

Scanning Electron Microscope (SEM)

Scanning Electron Microscope measurements were performed on a JEOL JSM-820 instrument.

Fluorescence Measurements

Fluorescent measurements were performed on a Middle-Range Spectrofluorometer FP-6300 from Jasco.

Fluorescent Microscopy

Images of cells were taken using a DM2500 Microscope from Leica coupled to a fluorescent spectrometer.

3. Cl-Sydnone synthesis and analytical data

The Cl-Syd was synthesized adapting the published procedure by Kolodych and Taran.[1]

Step 1: Synthesis of *p*-carboxyphenyl *N*-substituted α-amino acid

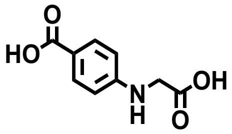

Sodium chloroacetate (6.99 g, 60 mmol) was dissolvent in 50 mL H_2O and vigorously stirred in an oil bath at 130 °C. Subsequently, the 4-aminobenzoic acid was added as solid (8.22 g, 60 mmol). The reaction was refluxed overnight. The solid product formed was filtered off and washed with cold H_2O. The recovered solid was extensively dried and subsequently recrystallized from ethanol. 7.8 g were isolated, resulting in a yield of 67%.

RP-HPLC (linear gradient from 0 - 50% eluent B in 20 min at 40 °C): tR = 5.65 min. Determined purity: 99%

ESI-MS for $C_9H_9NO_4$: (Exact monoisotopic mass 195.05): $[M-H]^-$ calcd. 194.05, found 194.3.

^{1}H NMR (250 MHz, DMSO-d_6): δ 12.38 (s, 2H), 7.67 (d, J = 8.8 Hz, 2H), 6.69 (s, 2H), 6.57 (d, J = 8.8 Hz, 2H), 3.87 (s, 2H).

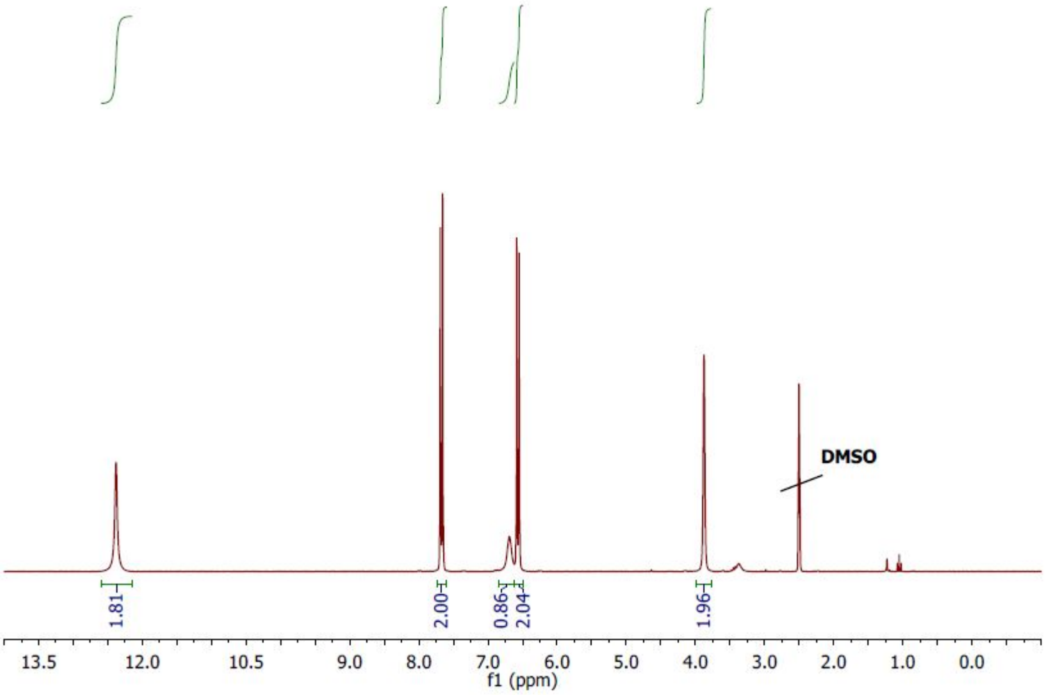

Figure S 1: ^{1}H-NMR (250 MHz, DMSO-d_6) of *p*-carboxyphenyl *N*-substituted α-amino acid.

Step 2: Synthesis of *N*-nitroso derivative of *p*-carboxyphenyl *N*-substituted α-amino acid

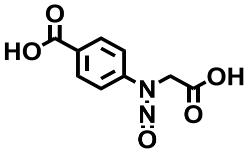

p-carboxyphenyl *N*-substituted a-amino acid (4.74 g, 24.3 mmol) was suspended in 20 mL aqueous HCl (10%) and stirred vigorously at 0 °C. To the stirred suspension, a solution of sodium nitrite (3.36 g, 48.6 mmol) was added slowly over a period of 60 min. The reaction was stirred overnight under nitrogen. The solid product was filtered off, washed with cold methanol and extensively dried. 5.24 g (23.4 mmol) of the nitroso intermediate were isolated which corresponds to a yield of 96%. The nitroso intermediate was used instantly in the next reaction step without purification. Remaining staring product was overserved during analysis, despite an increase in sodium nitrite.

RP-HPLC (linear gradient from 0 - 50% eluent B in 20 min at 40 °C): tR = 6.72 min. Determined purity: 84% (11.5 starting material with tR = 5.65 min).

ESI-MS for $C_9H_8N_2O_5$: (Exact monoisotopic mass 224.04): $[M-H]^-$ calcd. 223.04, found 223.4.

^{1}H NMR (250 MHz, DMSO-d_6): δ 13.58 (s, 1H), 8.28 – 8.14 (m, 2H), 8.13 – 8.01 (m, 2H), 7.90 (s, 1H).

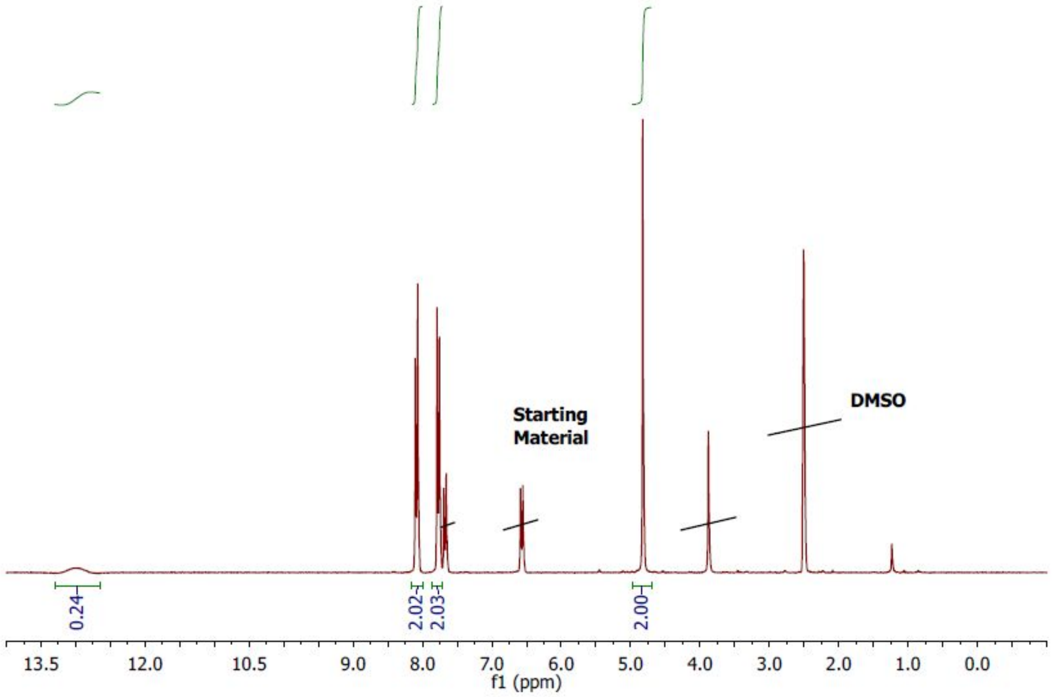

Figure S 2: ^{1}H-NMR (250 MHz, DMSO-d_6) of *N*-nitroso derivative of *p*-carboxyphenyl *N*-substituted α-amino acid

Step 3: Synthesis of *p*-carboxyphenyl-sydnone

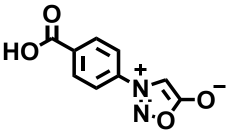

Baker-Ollis sydnone synthesis, dissolving the nitroso intermediate (5.24 g, 23.4 mmol) in 25 mL acetic anhydride and stirring the reaction at 110 °C for 4 h. Subsequently, half of the acetic anhydride is removed under reduced pressure and the yellow product formed during the reaction is filtered off and extensively dried. The product is recrystallized from methanol. 1.2 g (5.83 mmol) of *p*-carboxyphenyl-sydnone were isolated, which corresponds to a yield of 25 %.

RP-HPLC (linear gradient from 0 - 50% eluent B in 20 min at 40 °C): tR = 7.36 min. Determined purity: 99%

ESI-MS for $C_9H_6N_2O_4$: (Exact monoisotopic mass 206.03): $[M\text{-}H]^-$ calcd. 205.03, found 205.3.

^{1}H NMR (250 MHz, DMSO-d_6): δ 13.58 (s, 1H), 8.28 – 8.14 (m, 2H), 8.13 – 8.01 (m, 2H), 7.90 (s, 1H).

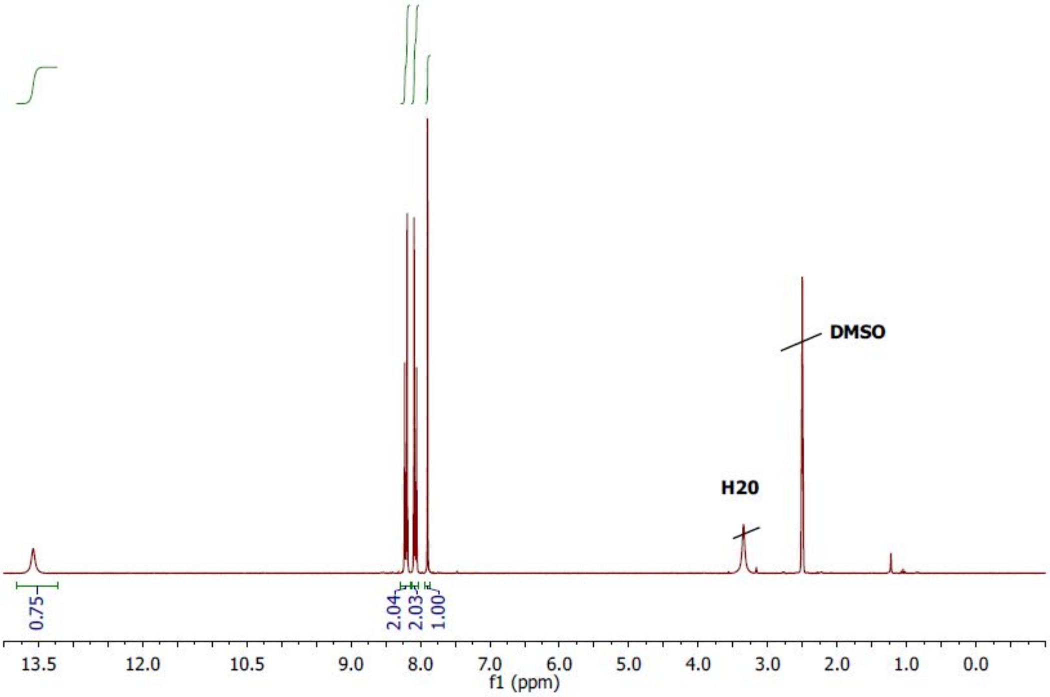

Figure S 3: ^{1}H-NMR (250 MHz, DMSO-d_6) of *p*-carboxyphenyl-sydnone

Step 4: Synthesis of *p*-carboxyphenyl-chlorosydnone

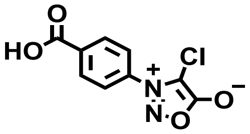

206 mg (1 mmol) of *p*-carboxyphenyl-sydnone were dissolved in 12 mL of a mixture of dioxane and HCL (1M) with a ratio of 2/1. 1.23 mL (1 mmol) of sodium hypochlorite (10 % solution in H_2O) were added dropwise. The reaction was stirred for 4 h. Subsequently, the reaction was quenched by pouring it into a solution of sodium thiosulfate (20 wt%). The product was extracted three times from the aqueous solution (27 mL in total) using 25 mL of ethyl acetate. The ethyl acetate was dried using sodium sulfate and subsequently removed under reduced pressure. 25.2 mg (0.10 mmol) were isolated after column chromatography using DCM/methanol 99/1 with 1 % acetic acid as eluent, corresponding to a yield of 10 %.

RP-HPLC (linear gradient from 0 - 50% eluent B in 20 min at 40 °C): tR = 9.36 min. Determined purity: 89%.

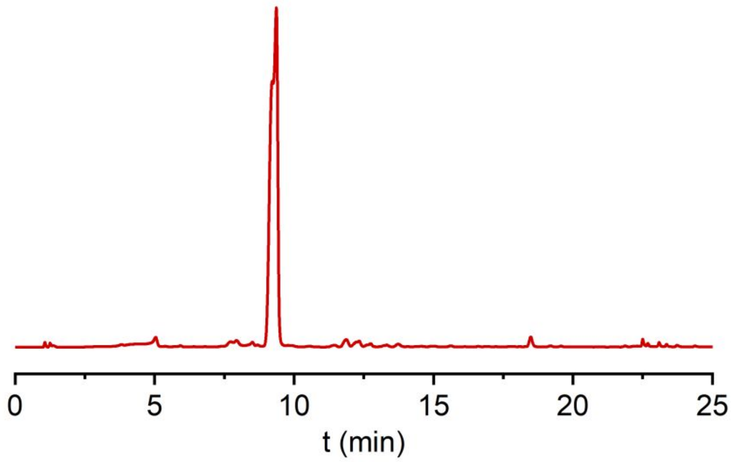

Figure S 4: RP-HPLC analysis (linear gradient from 0 - 50% eluent B in 20 min at 25 °C).

ESI-MS for $C_9H_5ClN_2O_4$: (Exact monoisotopic mass 239.99): $[M-H]^-$ calcd. 238.99, found 238.4.

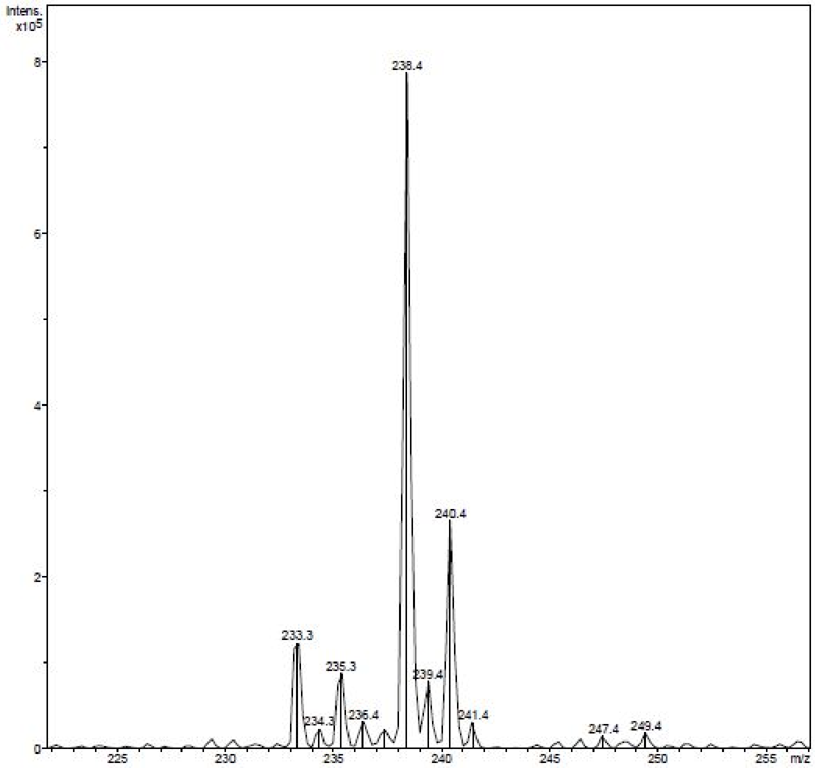

Figure S 5: ESI-MS analysis of *p*-carboxyphenyl-chlorosydnone.

^{1}H NMR (250 MHz, DMSO-d_6): δ 8.31 – 8.22 (m, 2H), 8.01 – 7.94 (m, 2H).

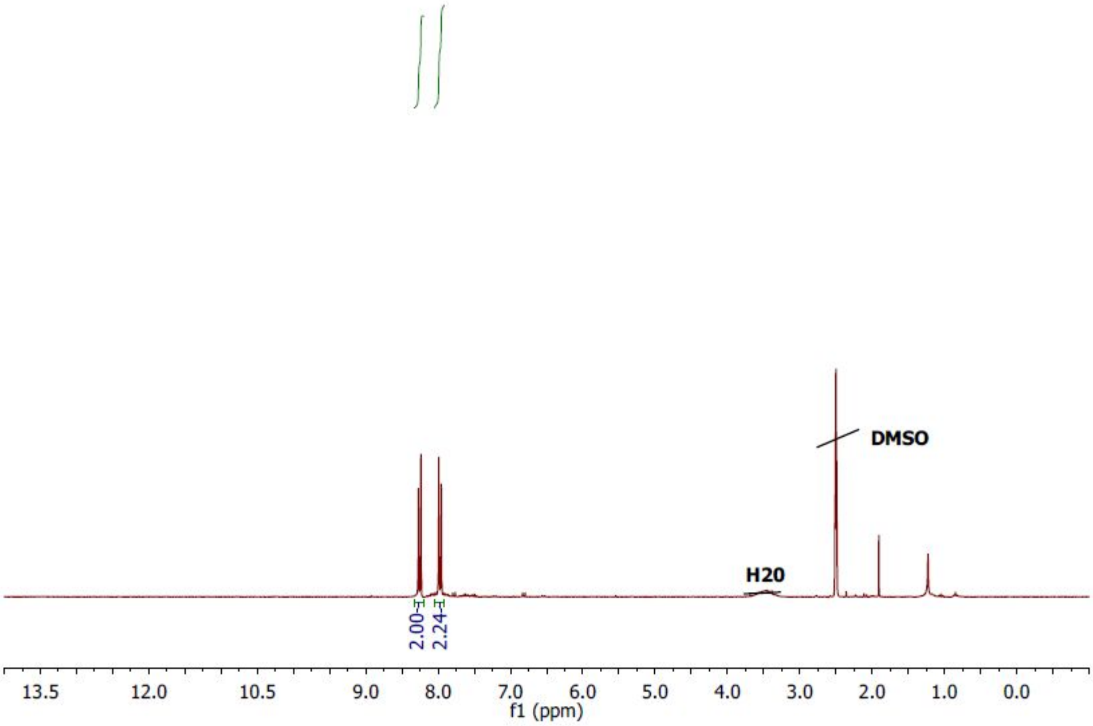

Figure S 6: ^{1}H-NMR (250 MHz, DMSO-d_6) of *p*-carboxyphenyl-chlorosydnone.

4. Kinetic NMR study

For the determination of the second order reaction rates, model compounds which were synthesized for a following project were applied, thereby making use of a more relevant compounds in terms of molecular weight and neighboring functional groups. Structures of the two compounds will not be revealed since they are parts of a study which is currently ongoing.

Sample preparation:

The Cl-Syd bearing compound was first measured at the same concentration and in the same solvents as subsequently applied in the kinetic study with the DBCO compound present. Therefore, 2 µmol of the CL-Syd bearing compound were dissolved in a mixture of 700 µL D_2O and 50 µL DMSO-d_6 (750 µL total, final concentration 2.66 mM) to measure the initial compound at the same conditions as subsequently during the kinetic study.

Following the measurement of the Cl-Syd-bearing compound, the kinetic with both compounds present was started. Therefore, the Cl-Syd derivative was filled into the NMR tube in 500 µL of D_2O and subsequently the DBCO derivative was added, dissolved in a mixture of 200 µL D_2O and 50 µL of DMSO-d_6. Final concentrations for the Cl-Syd and the DBCO derivative were 2.66 mM and 40 mM, respectively. NMR spectra was recorded after 60, 228, 368, 567, 759, 1193, 1771 and 2722 sec. NMR spectra of the initial Cl-Syd compound in the same solvent as used during the kinetic study as well as the spectra of the first five time points are shown below in Figures S 7 to Figure S 12.

NMR Spectra of kinetic measurement:

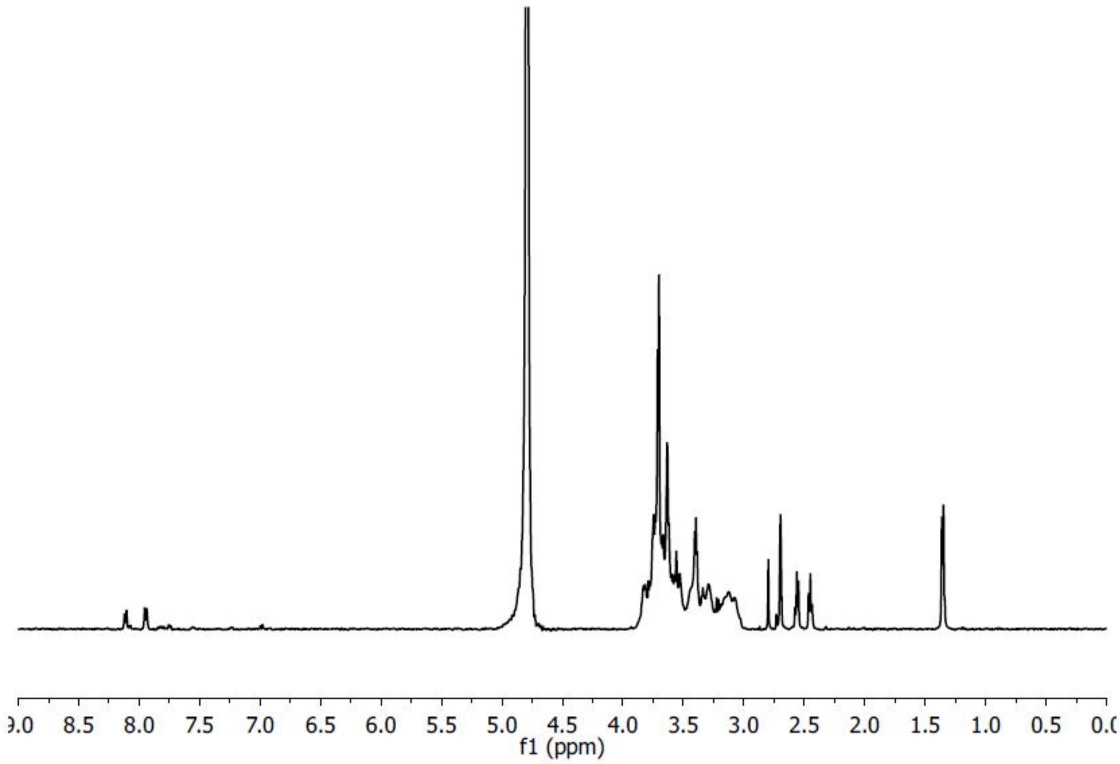

Figure S 7: ^{1}H-NMR (500 MHz, mixture of 700 µL D_2O and 50 µL DMSO-d_6) of *p*-carboxyphenyl-chlorosydnone at the same concentration and same solvent as in the following kinetic experiment. This spectrum was used as time point T = 0 sec.

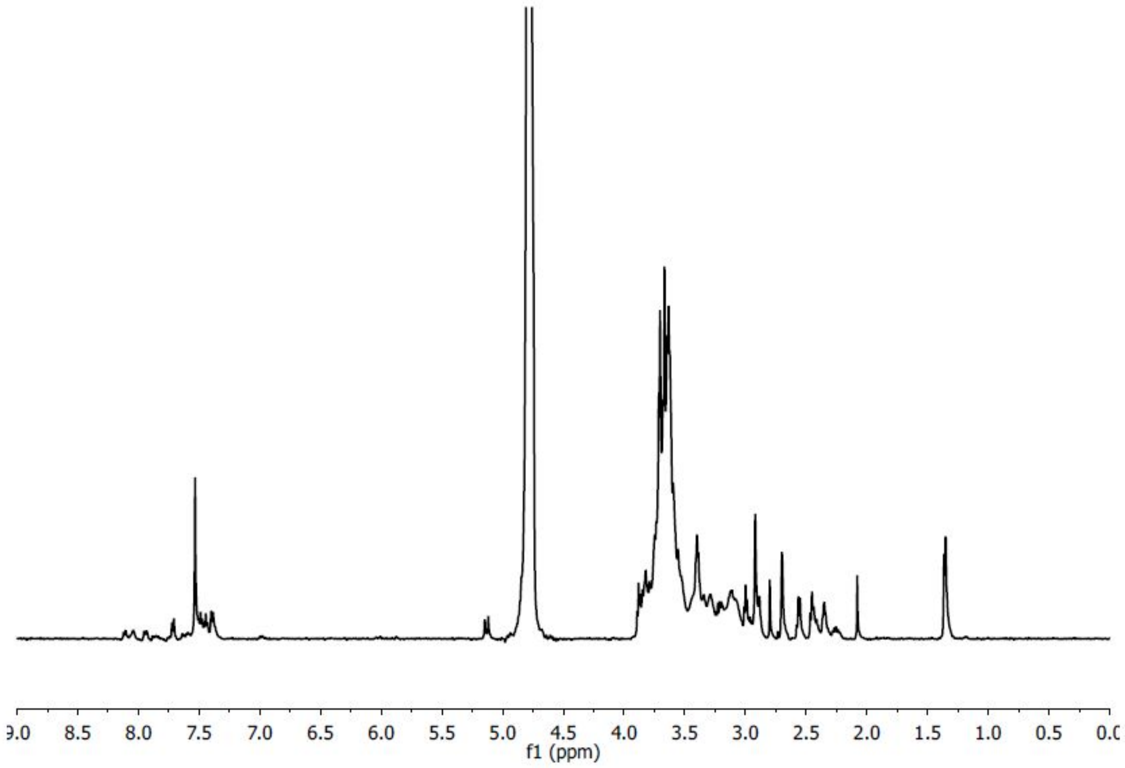

Figure S 8: ^{1}H-NMR (500 MHz, mixture of 700 µL D_2O and 50 µL DMSO-d_6) of the first time point T = 60 sec of the kinetic measurement between a DBCO and Cl-Syd derivative.

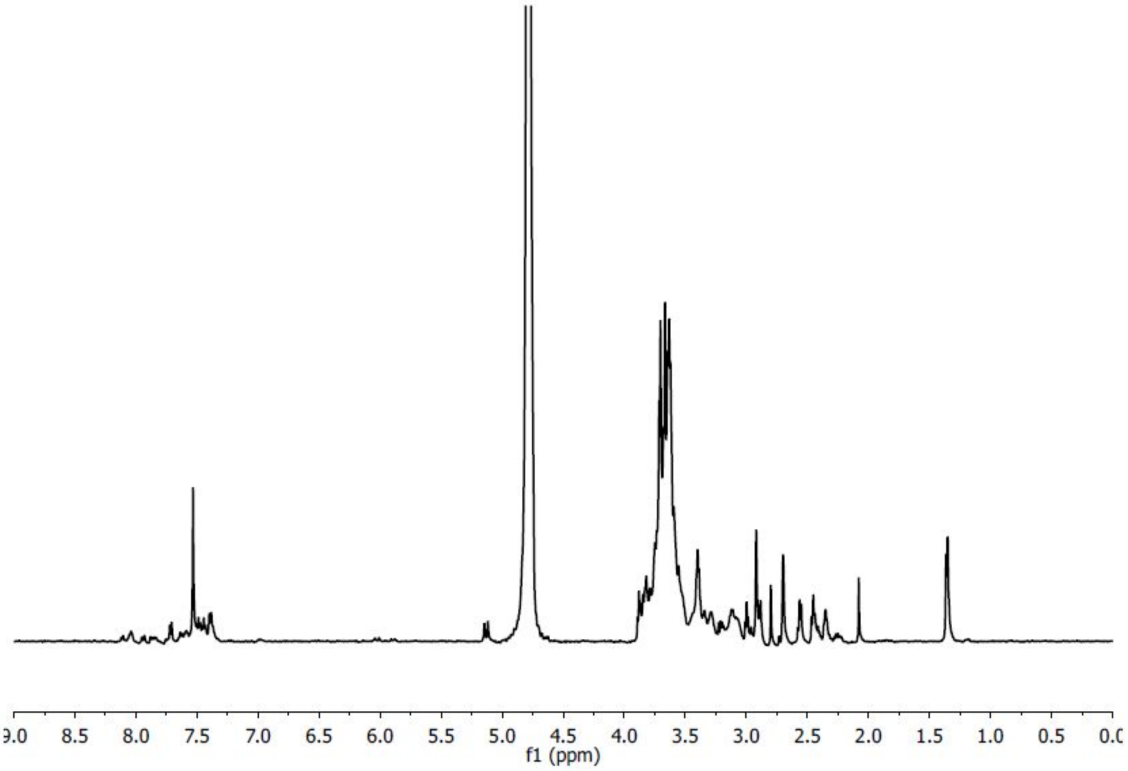

Figure S 9: ^{1}H-NMR (500 MHz, mixture of 700 µL D_2O and 50 µL DMSO-d_6) of the second time point T = 228 sec of the kinetic measurement between a DBCO and Cl-Syd derivative.

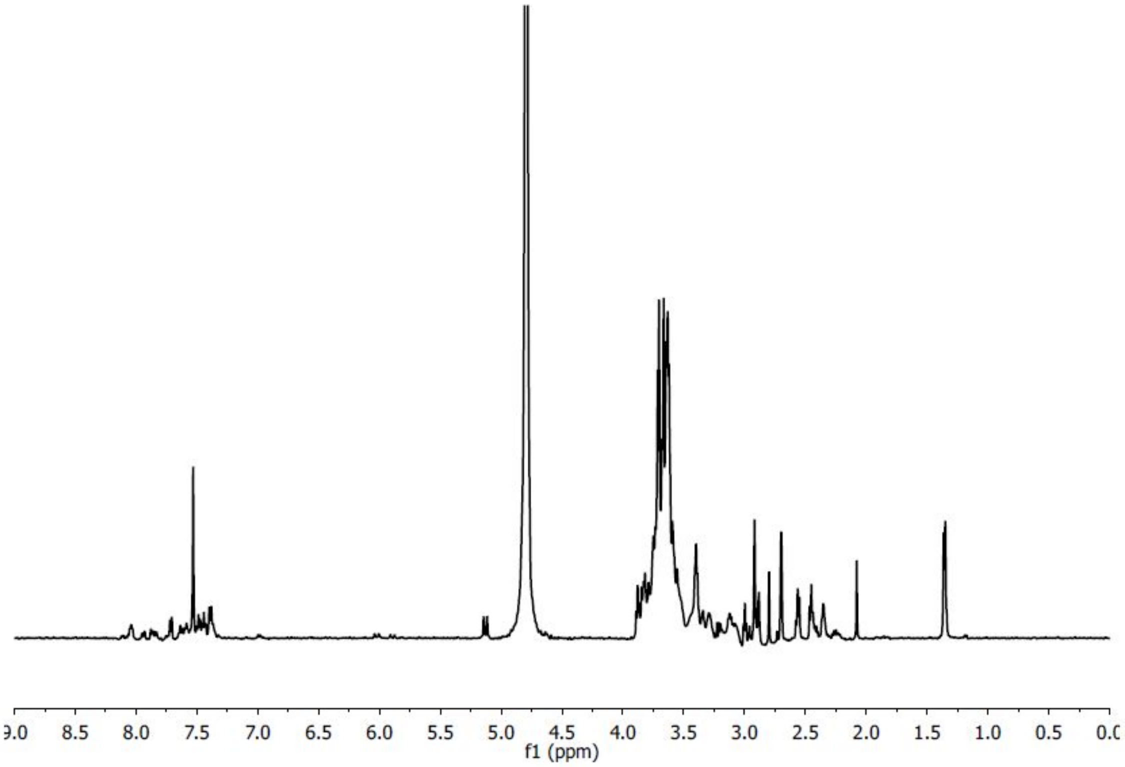

Figure S 10: ^{1}H-NMR (500 MHz, mixture of 700 µL D_2O and 50 µL DMSO-d_6) of the third time point T = 368 sec of the kinetic measurement between a DBCO and Cl-Syd derivative.

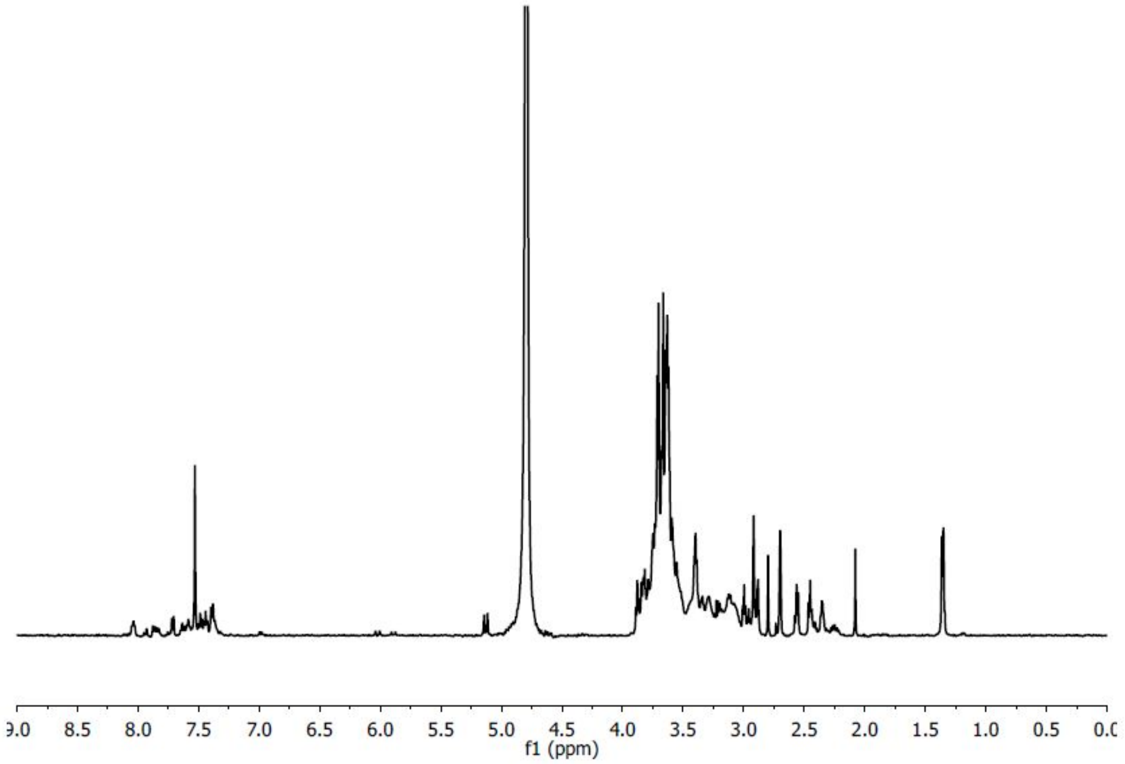

Figure S 11: ^{1}H-NMR (500 MHz, mixture of 700 µL D_2O and 50 µL DMSO-d_6) of the fourth time point T = 576 sec of the kinetic measurement between a DBCO and Cl-Syd derivative.

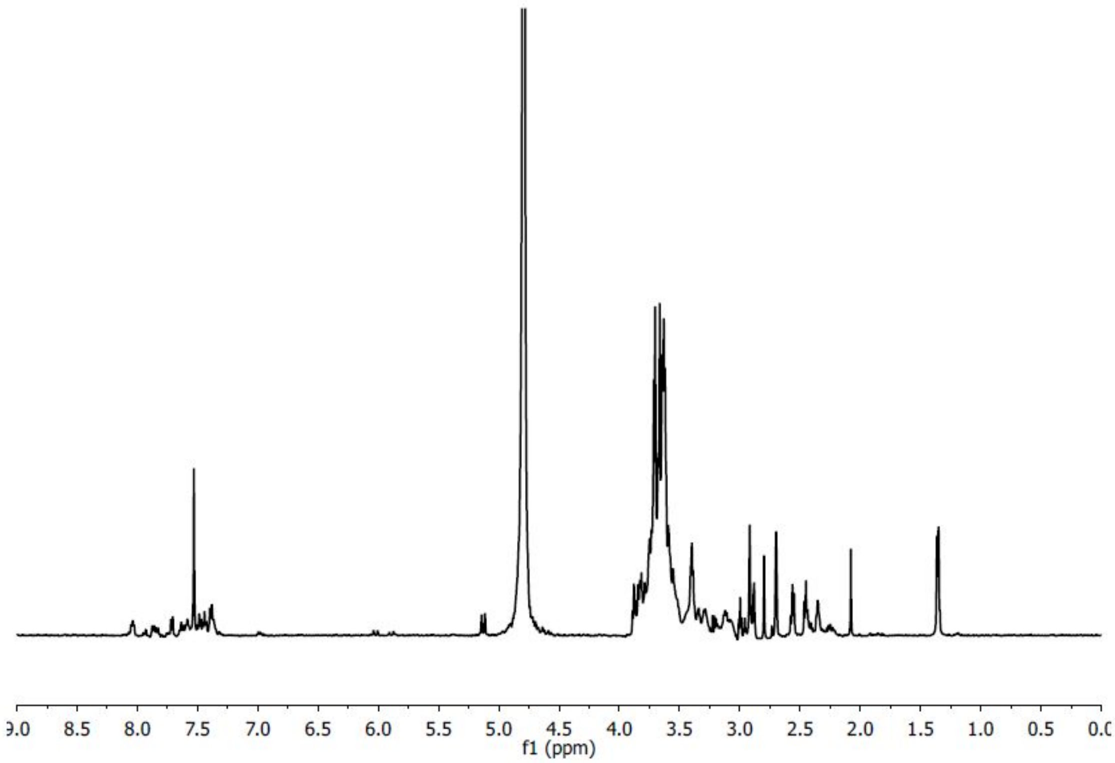

Figure S 12: ^{1}H-NMR (500 MHz, mixture of 700 µL D_2O and 50 µL DMSO-d_6) of the fifth time point T = 759 sec of the kinetic measurement between a DBCO and Cl-Syd derivative.

5. Synthesis of BSA NPs

The BSA NPs were synthesized by the desolvation methods, applying an established protocol from the group of Rubio-Retama.[2] In short, to a solution of 5 mg bovine serum albumin (BSA) in 1 mL of carbonate buffer (20 mM, pH of 8.5), 4 mL of ethanol were added at a flow rate of 3 mL/min. Upon addition of ethanol, the BSA NPs precipitated and 11.5 µL of glutaraldehyde (0.25 wt%) were added for BSA cross-linkage, thereby ensuring their stability.

6. Functionalization of BSA NPs with NHS activated Cl-Syd and FITC

To functionalize the BSA NPs with clickable Cl-Syd groups, 50 µL of an NHS activated Cl-Syd stock in DMSO (30 mM) were added to a suspension of BSA NPs at a concentration of 1 mg/mL in carbonate buffer (pH 8.5). The reaction was stirred overnight and subsequently purified by centrifugation. The BSA NPs were washed three times with carbonate buffer.
For FITC introduction, 50 µL of a FITC stock in DMSO (6.4 mM) were added to 1 mg/mL BSA NPs in carbonate buffer (pH 8.5). The reaction was allowed to proceed for 3 h at RT. FITC functionalized BSA NPs were purified by three washing steps using carbonate buffer.

7. Determination of Cl-Syd functionalization on BSA NPs

The amount of Cl-Syd functional groups per BSA-NP was calculated using the data obtained from the coupling assays with DBCO-functionalized sCy5 dye described in the paper (see Figure 2 in the paper). During the assay, four different concentrations of DBCO-sCy5 dye were applied, thereby proving the presence of Cl-Syd moieties on the BSA NPs surface. CS-BSA NPs were therefore incubated in solutions of DBCO-sCy5 with concentrations of 25, 50,100 and 250 µM. After incubation of the CS-BSA NPs in the solutions containing DBCO functionalized dye, supernatants were separated by centrifugation and the remaining amount of sCy5 dye was quantified. Thereby, it was determined, that 17 nmol of DBCO-sCy5 (85 µM as determined graphically, see Figure 2e) were required to completely functionalize the CS-BSA NPs. Since one DBCO reacts with one Cl-Syd moiety in the Strain-Promoted Sydnone–Alkyne Cycloaddition (SPSAC), the same amount of Cl-Syd is present in the reaction media, however, immobilized on the BSA NPs surface.
Therefore, an approximation of the concentrations of the BSA NPs in the reaction media was made, using the hydrodynamic radius of the BSA NPs prior to their functionalization (120 nm) for the calculations. For each coupling experiment, 0.1 mg of BSA NPs were used.

Determination of the amount of CS-BSA NPs per reaction:
At first, an approximation of BSA proteins per BSA NP was calculated. This was performed calculating the volume of one BSA protein (calculating with a diameter of 10 nm) as well the volume of the entire CS-BSA NP (calculating with the diameter of 120 nm). Volumes were calculated using the equation to determine the volume of a sphere. By dividing the volume of the BSA NP by the volume of one BSA protein, it can be assumed that each particle contains approximately 1.500 BSA proteins This is in accordance with the published value of albumin proteins per Abraxane® NP, which was stated to be 1757 for NPs (Aggregation number Z, determined by static light scattering experiments) with a slightly higher hydrodynamic radius of 130 nm.[3]
Considering the molecular weight of one BSA protein to be 60.000 Da, the molecular weight of one CS-BSA NPs can be approximated to be 99.000.000 Da. Since 0.1 mg of CS-BSA NPs were used in each click assay, approximately 1 pmol CS-BSA NPs were present ($6 \cdot 10^{12}$).

Determination of Cl-Syd moieties per CS-BSA NP:
Knowing the amount of CS-BSA NPs in each reaction to be 1 pmol and the fact that 17 nmol of DBCO-sCy5 were required to completely saturate all Cl-Syd moieties on the NPs surface, it can be concluded that each CS-BSA NP presents approximately 15.000 reactive Cl-Syd moieties.

8. anti-PD-L1 functionalization with clickable DBCO moieties

For the Ab functionalization, 5 µL of DBCO-PEG4-NHS stock (2.2 mM) in DMSO were added to a solution of 330 µg of anti-PD-L1 in 200 µL of PBS (pH 8.0) and incubated for 4 h at RT. After the coupling, the DBCO functionalized Abs were purified by centrifugal concentrators with a molecular weight cutoff (MWCO) of 50 kDa, that permits removing unreacted DBCO starting material.

9. References

(1) Plougastel, L.; Koniev, O.; Specklin, S.; Decuypere, E.; Créminon, C.; Buisson, D.-A.; Wagner, A.; Kolodych, S.; Taran, F. 4-Halogeno-Sydnones for Fast Strain Promoted Cycloaddition with Bicyclo-[6.1.0]-Nonyne. *Chem. Commun.* **2014**, *50* (66), 9376–9378.

(2) Arriagada, F.; Günther, G.; Zabala, I.; Rubio-Retama, J.; Morales, J. Development and Characterization of Florfenicol-Loaded BSA Nanoparticles as Controlled Release Carrier. *AAPS PharmSciTech 2019 205* **2019**, *20* (5), 1–8.

(3) Bhattacharyya, J.; Bellucci, J. J.; Weitzhandler, I.; McDaniel, J. R.; Spasojevic, I.; Li, X.; Lin, C. C.; Chi, J. T. A.; Chilkoti, A. A Paclitaxel-Loaded Recombinant Polypeptide Nanoparticle Outperforms Abraxane in Multiple Murine Cancer Models. *Nat. Commun. 2015 61* **2015**, *6* (1), 1–12.
